# High-Performance Gate-Controlled Superconducting Switches: Large Output Voltage and Reproducibility

**DOI:** 10.1021/acsnano.4c05910

**Published:** 2024-07-26

**Authors:** Leon Ruf, Elke Scheer, Angelo Di Bernardo

**Affiliations:** Department of Physics, University of Konstanz, Universitätsstraße 10, 78464 Konstanz, Germany

**Keywords:** niobium, superconductivity, critical current, gate tunability, superconducting nanobridges, three-terminal devices

## Abstract

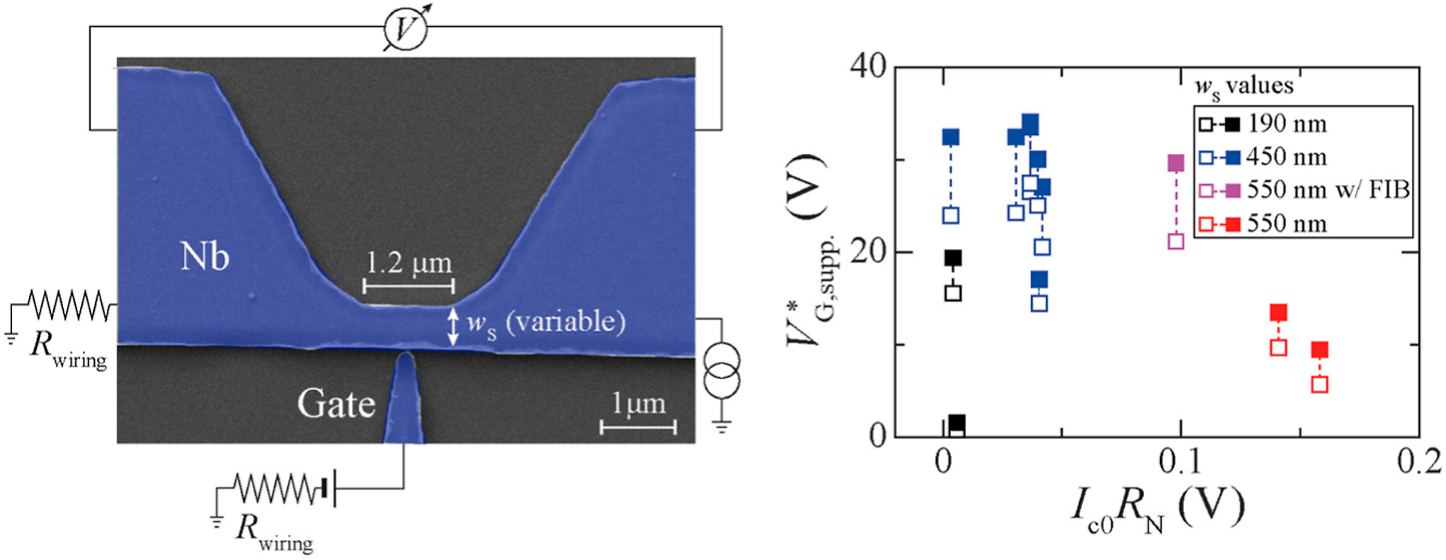

Logic circuits consist of devices that can be controlled between two distinct states. The recent demonstration that a superconducting current flowing in a constriction can be controlled via a gate voltage (*V*_G_)—gate-controlled supercurrent (GCS)—can lead to superconducting logic with better performance than existing logics. However, before such logic is developed, high reproducibility in the functioning of GCS devices and optimization of their performance must be achieved. Here, we report an investigation of gated Nb devices showing GCS with very high reproducibility. Based on the investigation of a statistically significant number of devices, we demonstrate that the GCS is independent of the constriction width, in contrast with previous reports, and confirm a strong correlation between the GCS and the leakage current (*I*_leak_) induced by *V*_G_. We also achieve a voltage output in our devices larger than the typical values reported to date by at least 1 order of magnitude, which is relevant for the future interconnection of devices, and show that *I*_leak_ can be used as a tool to modulate the operational *V*_G_ of devices on a SiO_2_ substrates. These results altogether represent an important step forward toward the optimization of reproducibility and performance of GCS devices, and the future development of a GCS-based logic.

The control of a superconducting current (supercurrent) via the application of a gate voltage (*V*_G_), currently known as gate-controlled supercurrent (GCS), has become a subject of great interest, as evidenced by the number of experimental^1−29^ and theoretical studies^30−36^ reported on it. Among the main motivations behind the interest in the GCS is its potential for the development of voltage-controlled superconducting logics that would intrinsically have low-energy dissipation (since based on superconductors) and better performance than other superconducting logics already available.^37−39^ Compared to these, GCS-based logics would offer several advantages including higher device density (due to the smaller device size), larger number of devices connectable in series (i.e., higher fan out), stronger resilience to magnetic noise and easier interfacing with conventional metal-oxide semiconductor (CMOS) circuits to form hybrid superconducting/semiconducting computing architectures.^40^ Also, superconducting GCS devices can find applications in emerging quantum technologies, and particularly in superconducting quantum processing units (QPUs). As recently suggested,^40^ GCS devices could be integrated into QPUs and used, for example, as tunable elements to switch on/off the control of multiqubit gates. In addition, these devices could be integrated also into auxiliary systems (filters, resonators etc.) for the multiplex routing of microwave signals to the QPU.

To develop some of the above applications based on the GCS, however, it is first necessary to overcome fundamental challenges and to meet proper technological standards, both in terms of performance of the individual GCS-based devices and in terms of optimization of their fabrication process.

Some of the current fundamental challenges stem from the lack of a clear understanding of the mechanism responsible for the GCS, which is in turn crucial to achieve control over the effect. As reported in a recent review on the GCS,^40^ four main mechanisms have been proposed to date to explain the GCS. Some of these mechanisms ascribe the GCS to phenomena triggered by the finite leakage current (*I*_leak_) that flows in most device realizations between the gate and the superconductor (S) constriction under an applied *V*_G_. These phenomena include tunneling of high-energy electrons between the gate and the S,^12−14,23,24^ phonons induced by *I*_leak_ in the substrate heating the S constriction,^21^ and high-energy electrons or phonons induced by *I*_leak_ which drive the S into an out-of-equilibrium state characterized by phase fluctuations but without heating.^19,22^ In addition to these *I*_leak_-related mechanisms, it has also been suggested that the electric field (associated with the applied *V*_G_) can induce effects responsible for a GCS.^1−10,15,18,25^

It is worth noting that the diversity in the mechanisms proposed to explain the GCS partly originates also from the significant differences in materials (i.e., S type, substrate etc.), device geometry and experimental setups used in the different studies, which makes it difficult also to find a “universal mechanism” at play in all studies on GCS. At this stage, it is therefore crucial to carry out studies where all material and device parameters are kept fixed, and only one parameter at a time is systematically varied, to rule out and/or confirm the main findings made in other reports.

In terms of performance, the voltage *V*_G,offset_ needed to induce a full suppression of the critical supercurrent (*I*_c_) and switch a GCS device to its normal state, must be reduced to few volts, to better interface GCS devices with CMOS devices (typically working at *V*_G_ < 5 V; ref (41)) in hybrid computing platforms. A lower *V*_G,offset_ would also lead to an increase in the fan out, since the output voltage *V*_out_ of a GCS device, which depends on its characteristic voltage *I*_c0_*R*_N_ (*R*_N_ being the normal-state resistance and *I*_c0_ being *I*_c_ at *V*_G_ = 0), can be fed as input signal to the gate (i.e., used as the *V*_G_) of another GCS device connected downstream.^40^ In most GCS devices reported to date, *V*_G,offset_ is typically of few tens of volts,^1,2,6,9,10,17,25,27^ while *I*_c0_*R*_N_ is typically of few millivolts or tens of millivolts,^1,2,4,6,15,20,22,27^ meaning that these two voltages differ by several orders of magnitude. This large difference between *I*_c0_*R*_N_ and *V*_G,offset_ currently makes the connection of GCS devices in series not feasible.

In terms of the fabrication process, it is necessary to optimize protocols for high scalability of devices and to achieve high reproducibility in their realization of a GCS. Several groups have already tried to scale up the fabrication of GCS devices using subtractive patterning,^23,26,27^ which involves lithographic patterning of a device into a negative resist layer used then as mask to etch an underlying S thin film, other than by additive patterning involving lithographic patterning of a polymer mask followed by S material growth and lift off. Some of these studies, however, suggest that devices made by subtractive patterning do not show a GCS unlike devices made by additive patterning,^26^ unless unconventional Ss that can be grown with a small grain size (e.g., Nb_0.18_Re_0.82_) are used, in combination with a nontrivial surface chemistry activated by the etching process.^27^

Achieving high reproducibility in the behavior of GCS-based devices, which is also essential for a better fundamental understanding of the effect as explained above, remains another crucial objective. All the studies on the GCS reported to date are in fact based on the characterization of few devices (typically one or two devices in each study), across which several parameters (e.g., substrate, S type, gate-to-channel distance, device geometry) are often varied, even within the same study.^40^ This large variation of parameters makes it difficult to agree on the existence of universal features of the GCS, since certain observations are not reproduced when GCS devices with different parameters are studied.

Here, we address some of the open challenges listed above in the field of GCS. First, we report a fabrication process that allows to systematically reproduce the phenomenon in all our investigated (13) devices. The high reproducibility achieved also allows us, by varying one parameter at a time across different devices, to confirm or demystify sporadic observations, which are often based on a less statistically relevant number of samples. For example, by progressively varying the width of our S constriction (*w*_S_), while keeping the other device parameters identical, we show that GCS does not require *w*_S_ of the same order of magnitude as the S coherence length for it to be observed, in contrast with previous observations.^1,13^ Thanks to an in-depth analysis of other performance parameters of our devices, we also rule out *I*_leak_-induced Joule heating as possible mechanism for the GCS but confirm the existence of a strong correlation between the suppression of *I*_c_ and variations in *I*_leak_, consistent with other reports.^28^

Second, the large *w*_S_ used (up to 550 nm) results in devices with *I*_c0_*R*_N_ up to ∼0.25 V at 1.5 K, which represents a significant step forward toward the increase in the voltage output, as described above. Last, concurrently with an increase in *I*_c0_*R*_N_, we show that stress induced in the SiO_2_/Si substrate by *I*_leak_ can be used as a tool to reduce *V*_G,offset_. This result does not only possibly explain differences reported in *V*_G,offset_ even across nominally identical devices and measured under the same conditions,^2,22^ but it also provides a possible route to explore for the control of the operational *V*_G,offset_ of a device after its fabrication.

## Results and Discussion

**Figure 1:**
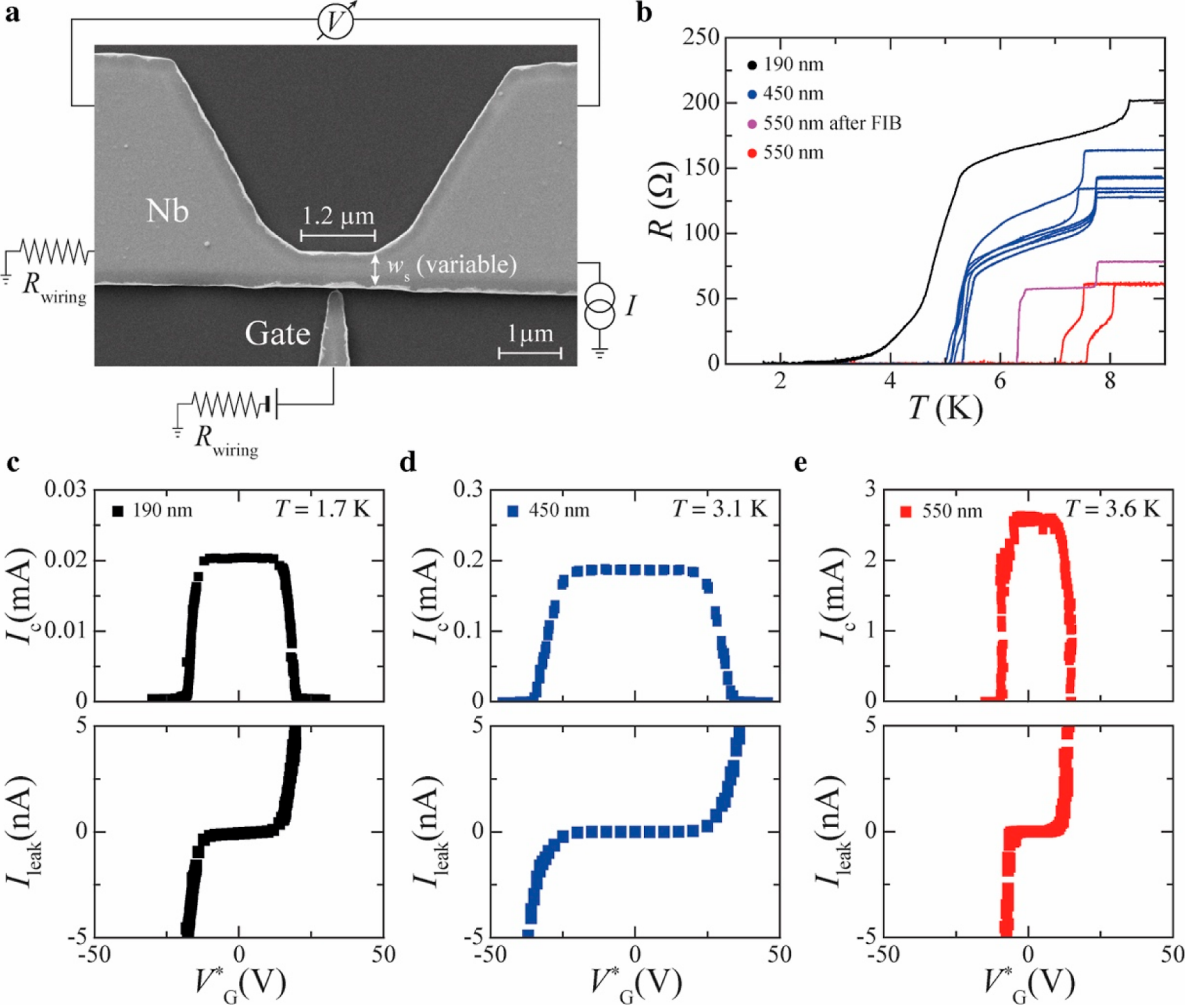
GCS in Nb Dayem bridges with different width. (a) Scanning electron micrograph of a typical gated Nb Dayem bridge with a schematic of the setup used for a four-point measurement of its electronic transport properties. (b) Resistance versus temperature *R*(*T*) measured for a few representative Nb Dayem bridges with width *w*_S_ of 190 nm (black; 1 device), 450 nm (blue; 6 devices), 550 nm without (red; 2 devices) and with 90 nm-wide cut made by FIB on the S constriction opposite to the gate (purple; 1 device). (c,d) Critical current versus applied effective gate voltage *I*_c_(*V*_G_^*^) (top panels) and corresponding leakage current versus *V*_G_^*^, *I*_leak_(*V*_G_^*^), curves (bottom panels) measured for a Dayem bridge with *w*_S_ equal to 190 nm (c), 450 nm (d), and 550 nm (e). The measurement *T* is specified in the top-right corner of each panel. *V*_G_^*^ is defined as *V*_G_^*^ = *V*_G_ – *I*_c_·*R*_wiring_ where *I*_c_·*R*_wiring_ is the additional voltage drop induced by a bias current *I*_bias_ = *I*_c_ passing through the wiring resistance *R*_wiring_ as illustrated in (a).

To investigate the reproducibility of the GCS in three-terminal superconducting devices, we have fabricated a series of gated Nb Dayem bridges (Figure 1a) on a SiO_2_ (300 nm)/undoped Si substrate. Across our devices, we have kept all design parameters fixed, except for *w*_S_. The parameters that we have kept fixed include the thickness of the S layer (27 nm-thick Nb deposited onto ∼4 nm of Ti used to promote adhesion), the shape of the gate (pointy), the gate distance from the Nb nanoconstriction (∼50 nm), and the length of the Nb nanoconstriction (∼1.2 μm). For *w*_S_, we have changed its value from 190 to 550 nm across our devices. The list of all the devices investigated in this study with the corresponding geometry and other experimental parameters obtained from their low-temperature characterization is reported in Table S1 of the Supporting Information.

Figure 1a shows a scanning electron microscope image of one of the Nb Dayem bridges with a sketch of the four-terminal probe setup used to measure the voltage versus current, *V*(*I*), characteristics, while applying a *V*_G_ to the side-gate electrode. In our setup, the actual gate voltage applied, hereby named *V*_G_^*^, is not simply equal to the *V*_G_ applied by the source meter (see Methods). Since the gate electrode and the negative terminal (*I*^–^) of the bias current, *I*_bias_, are connected to the same electrical ground (Figure 1a), *V*_G_ is shifted from *V*_G_^*^ by the voltage drop on the wiring resistance (*R*_wiring_), meaning that *V*_G_^*^ = *V*_G_ – *I*_bias_·*R*_wiring_ (Figure 1a). Due to the large *I*_bias_ that we apply in our devices to match *I*_c_ (up to 3 mA), the shift in *V*_G_ can be as large as 6 V (see Supporting Information). In the following, we therefore show the actual *V*_G_^*^ applied to the S constriction, after correcting for the voltage drop on *R*_wiring_ when *I*_bias_ = *I*_c_.

The data reported in Figure 1b–e for a few representative devices with different *w*_S_ show that we have achieved a systematic observation of a full superconducting transition and of the GCS in our devices (see also Table S1 and Figure S1 in the Supporting Information). We have investigated a total of 13 samples with a superconducting critical temperature (*T*_c_) ranging from 4.4 K for *w*_S_ = 190 nm to 7.57 K for *w*_S_ = 550 nm (Figures 1b and S1 in the Supporting Information), and they all show a GCS. In this paper, we define *T*_c_ as the temperature *T*, where the device resistance *R* reaches 10% of its normal-state (*R*_N_) value at 10 K. This definition yields lower *T*_c_ values than those given by more common *T*_c_ definitions based on 90%-*R*_N_ or 50%-*R*_N_ criteria. The reason why we have chosen this definition is related to the appearance of multistep transitions in the resistance versus temperature, *R*(*T*), characteristics that can be attributed to the lower thickness of the constriction (resulting from the sputtering deposition of Nb) compared to the thickness of the lateral wider areas. We have also made devices where we have cut the S constriction (on the side opposite to the gate) using a focused ion beam (FIB) with Ga^+^ ions, to assess the role of current-crowding^42,43^ effects on the GCS observation.

Most of the samples have been deliberately made with *w*_S_ much larger than the typical *w*_S_ (up to ∼200 nm) reported in previous studies on the GCS.^1,3−10,12,20,22,25,27,28^ This has been done to verify the argument that a GCS is easier to observe as *w*_S_ approaches the coherence length ξ of the S. Our measurement results instead show that GCS can also occur in devices with *w*_S_ of 550 nm (Figure 1e), which is ∼40 times larger than the ξ of Nb in the diffusive regime (ξ < 15 nm; ref (44)). We have not tested devices with larger *w*_S_, which may in principle still show GCS. We also observe that the gate voltage needed to suppress *I*_c_ does not increase as *w*_S_ gets larger (Figure 1c–e). These observations altogether suggest that whichever the mechanism responsible for the GCS in our devices is, such mechanism does not get suppressed when *w*_S_ greatly exceeds ξ.

Our results also imply that GCS does not require a weak superconducting constriction with small *I*_c_ to occur. This is crucial for future technological applications of GCS-based devices because, although a reduction in the device lateral dimensions is helpful to increase device density, wider constrictions ensure better device stability against prolonged device operation and thermal cycling.

We note that there is only one report to date^24^ about the observation of a GCS in micrometer-wide Nb bridges (i.e., with *w*_S_ ≫ ξ), but these devices have a top-gate other than a side-gate geometry, unlike the ones investigate in the present study. In a device with a top gate, the most relevant dimension for the GCS is not *w*_S_ but the S thickness, which in ref (24) is ∼6 nm and hence still comparable to ξ (<15 nm; ref (44)).

### Variation in the GCS with Constriction Width

The high reproducibility that we have achieved in the observation of a GCS across our devices is not only important as proof-of-principle for future technological applications of the GCS. This reproducibility also allows, by systematically varying only one parameter at a time across different devices, to test whether (1) such parameter is crucial for the GCS observation and (2) if any choices of this parameter improve the device performance (e.g., by reducing the voltage *V*_G,offset_^*^ needed for a full *I*_c_ suppression).

In this study, as also illustrated in Figure S2 of the Supporting Information, we define *V*_G,onset_^*^ and *V*_G,offset_^*^ as the values of *V*_G_^*^ at which *I*_c_ has dropped by 10% and 90%, respectively, compared its value at *V*_G_^*^ = 0 (i.e., *I*_c0_). All our devices show a *V*_G,offset_^*^ between ∼9.5 V and ∼34 V (Figure 1c–e and Table S1 of the Supporting Information). Only in one device we have measured a much smaller *V*_G,offset_^*^ (∼1.6 V), possibly because the *I*_leak_ measured at *V*_G,offset_^*^ for this device is larger by 1 order of magnitude than that typically measured in the other devices (Table S1 of the Supporting Information).

**Figure 2:**
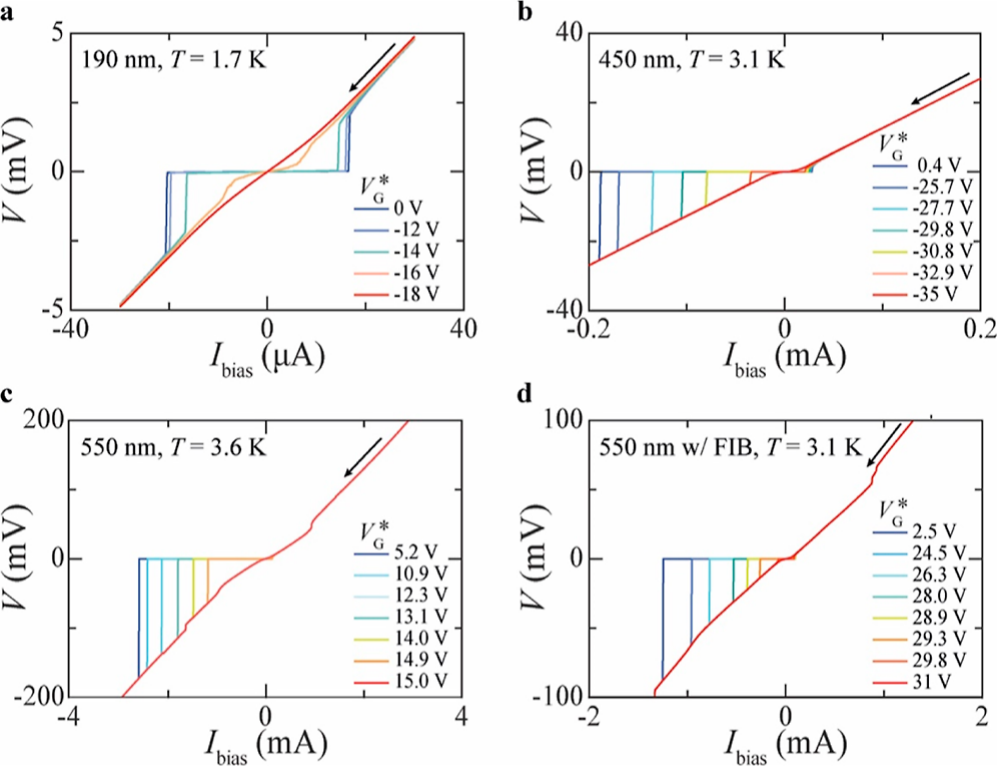
GCS for different device widths. (a–d) Voltage versus current, *V*(*I*), characteristics as a function of the applied effective gate voltage *V*_G_^*^ for different S constriction widths *w*_S_: 190 nm (a), 450 nm (b), 550 nm (c) and 550 nm after 90 nm-wide cut made by FIB opposite to the gate electrode. All the *V*(*I*) curves shown are measured while decreasing *I*_bias_ as indicated by the black arrow in each panel. In each panel, the *V*_G_^*^ values are defined at *I*_bias_ = *I*_c_, and the measurement temperature is specified in the top-left corner.

In Figure 2, we show the *V*(*I*) characteristics measured at a few representative *V*_G_^*^ for devices with different *w*_S_ showing a GCS. Figure 2c, for example, shows that a device with the largest *w*_S_ = 550 nm and hence largest *I*_c0_ (∼2.57 mA at *T* = 3.6 K) has smaller *V*_G,offset_^*^ compared to a device with *w*_S_ = 450 nm and *I*_c0_ = 0.187 mA at *T* = 3.1 K (Figure 2b). In general, we find that the dependence of *V*_G,offset_^*^ on *w*_S_ is not monotonic.

The *V*(*I*) curves at different *V*_G_^*^ in Figure 2 show that *V*_G,offset_^*^ increases when going from *w*_S_ = 190 nm (Figure 2a) to *w*_S_ = 450 nm (Figure 2b), and then decreases again from *w*_S_ = 450 nm (Figure 2b) to *w*_S_ = 550 nm (Figure 2c). Also, if a device with original width *w*_S_ = 550 nm is reduced to *w*_S_ ∼ 460 nm by FIB cutting, its *V*_G,offset_^*^ can even increase (Figure 2d) compared to the *V*_G,offset_^*^ measured for a twin device (i.e., made in the same batch) with *w*_S_ = 550 nm that has not been FIB-cut (Figure 2c).

Below, we show that the presence of conducting paths in the SiO_2_/Si substrate can induce variations in *V*_G,offset_^*^. Since the location of these conducting paths cannot be controlled or determined a priori and their formation also depends on the device measurement history, these effects can account for the apparent serendipity observed across our devices in their *V*_G,offset_^*^ dependence on *w*_S_.

### Independence of the GCS on Current Crowding

In devices with sharp edges between the S constriction and the lateral pads, current-crowding effects may appear,^42,43^ which can in turn affect the GCS. In our Nb Dayem bridges, we have tried to minimize current-crowding effects by deliberately fabricating devices with rounded corners at the intersection between the S constriction and the lateral pads (Figure 1a). The term current-crowding denotes the observation that sharp edges and sudden changes of the cross section result in a locally enhanced current density, which can in turn locally overcome the critical current density of *S*.

To investigate whether current crowding plays any role in the GCS of our devices, we have also deliberately introduced a sharp edge in one of our devices around which current-crowding effects should become more prominent. This sharp edge has been made by cutting out part of the S constriction using FIB, after the deposition and lift-off of Nb (see Methods). As shown in Figure S3 in the Supporting Information, the FIB cut (∼90 nm in width and ∼80 nm in length) has been made on a device originally with *w*_S_ = 550 nm on the constriction side opposite to the gate. This has been done to minimize Ga^+^ implantation in the channel between gate and constriction, which may lead to an increase in the *I*_leak_ and in turn affect the measured *V*_G,offset_^*^.

The measurement data in Figures 2d and S3 in the Supporting Information show that, although *I*_c0_ is reduced for the FIB-cut device by almost 50% compared to the twin uncut device (due to the *w*_S_ reduction and possible FIB-induced damage), *V*_G,onset_^*^ and *V*_G,offset_^*^ have increased by more than 250% after the FIB cut (see devices D10 and D12 in Table S1 in the Supporting Information). This result per se may suggest that current crowding does not foster or support the GCS. However, as shown below, a better parameter to compare different devices than *V*_G,offset_^*^ is the power dissipated by the gate in the device at *V*_G_^*^ = *V*_G,offset_^*^, namely *P*_G,offset_^*^ = *V*_G,offset_^*^I_leak,offset_ (with *I*_leak,offset_ being *I*_leak_ measured at *V*_G_^*^ = *V*_G,offset_^*^). Unlike *V*_G,offset_^*^, *P*_G,offset_^*^ is independent of the history of the device in terms of current-induced stress.

**Figure 3:**
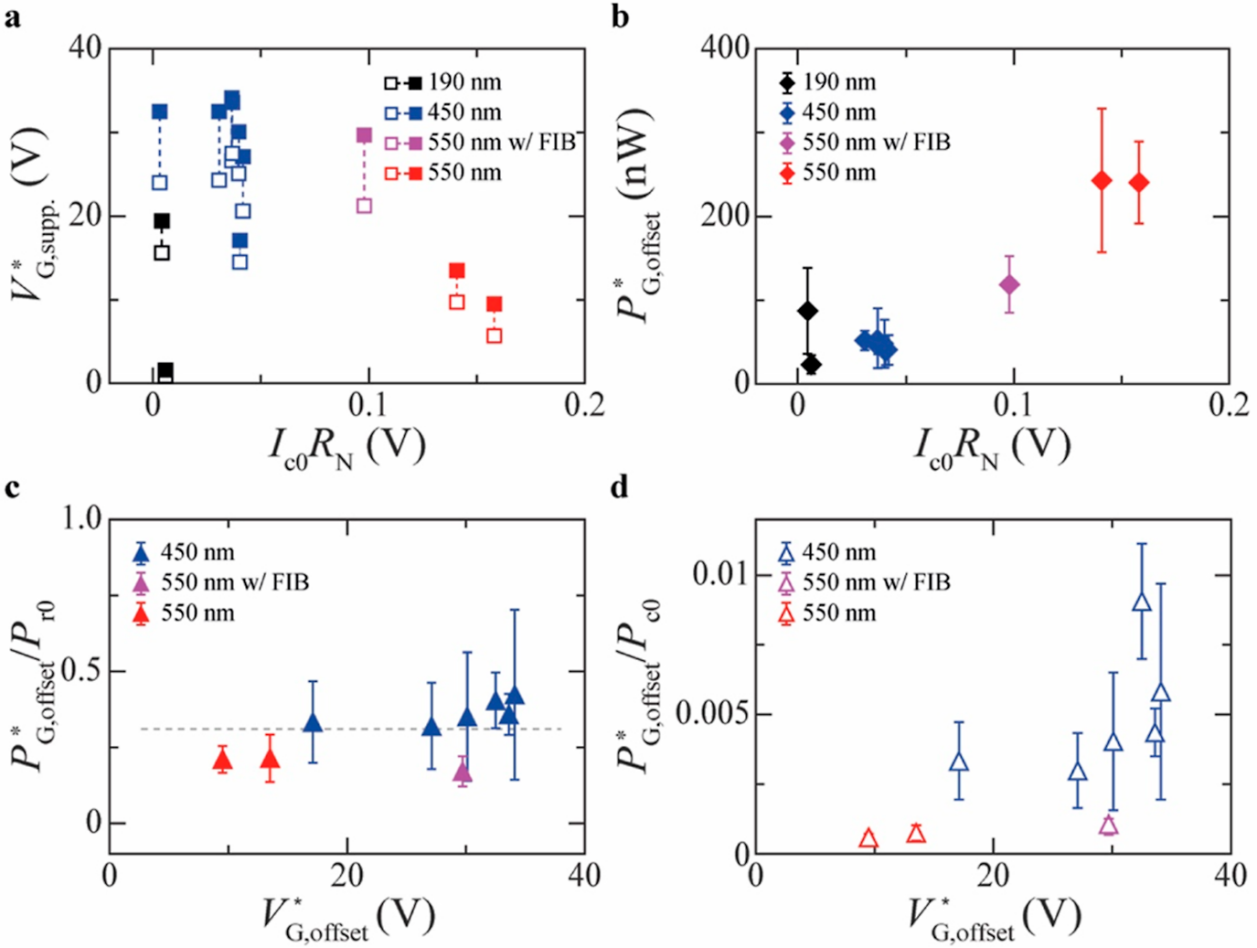
Figures of merit of Nb Dayem bridges. (a) Gate voltage needed to suppress *I*_c_, *V*_G,supp._^*^, at 10% of its value *I*_c0_ at *V*_G_^*^ = 0 (*V*_G,onset_^*^; empty squares) and at 90% of *I*_c0_ (*V*_G,offset_^*^; full squares) as a function of *I*_c0_*R*_N_ for devices with different widths *w*_S_ (specified in the legend). (b) Power dissipation induced by the gate voltage at *V*_G,offset_^*^, *P*_G,offset_^*^, as a function of *I*_c0_*R*_N_ for different *w*_S_ values (specified in the legend). (c, d) Ratio between *P*_G,offset_^*^ and *P*_r0_ = *R*_N_*I*_r0_^2^ (c) and between *P*_G,offset_^*^ and *P*_c0_ = *R*_N_*I*_c0_^2^ (d) (*I*_r0_ being the retrapping current at *V*_G_^*^ = 0) as a function of *V*_G,offset_^*^ for devices with different *w*_S_ values (specified in the legend). The data points in panels from (b–d) refer to devices all measured in the same setup.

In Figure S4 of the Supporting Information we show the *I*_c0_*R*_N_/*P*_G,offset_^*^ ratio for all devices with different *w*_S_ measured in the same cryostat, where contributions to *I*_leak,offset_ from the setup (e.g., due to the shielding of the wires), and in turn to *P*_G,offset_^*^, can be assumed constant. By determining the ratio *I*_c0_*R*_N_/*P*_G,offset_^*^, we normalize changes in *I*_c0_*R*_N_, due to the different *w*_S_ of our devices (see also Figure 3a), to the power needed to fully suppress *I*_c0_ in each device. If current crowding played a significant role toward the GCS in device D10 after the FIB cut, we would expect that the *I*_c0_*R*_N_/*P*_G,offset_^*^ ratio gets much higher for device D10 compared to the device D12 because current crowding should lead to the GCS observation at a lower *P*_G,offset_^*^. However, the change in *I*_c0_*R*_N_/*P*_G,offset_^*^ from device D12 to device D10 is of the same order in magnitude as that measured when comparing device D12 to other devices with smaller *w*_S_ yet equal to that of D10 (see blue data points in Supporting Information Figure S4), but with intentionally made current-crowding points. Therefore, although we cannot fully exclude that current crowding induces a small change in the *I*_c0_*R*_N_/*P*_G,offset_^*^ ratio for device D10, which falls within our measurement resolution for *P*_G,offset_^*^, our analysis suggests that current crowding does not play a significant role toward the GCS in this FIB-cut device.

### Large Output Voltage and Other Figures of Merit

As described above, another challenge for technological applications of the GCS is to increase the output voltage *V*_out_ of a GCS device when this is switched to the normal state by an applied *V*_G,offset_^*^, so that *V*_out_ > *V*_G,offset_^*^ also for a second device connected downstream. In addition to decreasing *V*_G,offset_^*^, another way to meet this condition is through increasing *V*_out_, which in turn depends on the characteristic voltage *I*_c0_*R*_N_ of a GCS device.

The *I*_c0_*R*_N_ values of the GCS devices reported to date are typically in the range from few millivolts to tens of millivolts, as reported in a recent review on GCS.^40^ Thanks to the larger *w*_S_ compared to those investigated before, our devices also exhibit larger *I*_c0_*R*_N_ values. Figure 3a shows that we have obtained *I*_c0_*R*_N_ larger than 0.15 V for devices with *w*_S_ = 550 nm. We have measured *I*_c0_*R*_N_ ∼ 0.16 V at *T* = 3.6 K, which is half the *T*_c_ of the device (∼7.1 K), and *I*_c0_*R*_N_ ∼ 0.25 V when the same device (device D11) was cooled down to ∼1.5 K.

Considering that no indication of suppression or saturation of the GCS was observed up to the maximum *w*_S_ > 550 nm investigated here, we believe that *I*_c0_*R*_N_ values up to 1 V are within reach, e.g. when using even wider or longer constrictions. Also, even keeping the same device geometry used in this study but replacing Nb with another S like NbN or NbRe with higher normal-state resistivity ρ_Ν_ and/or higher critical current density *J*_c_, can help increase *I*_c0_*R*_N_ up to or above 1 V, especially at measurement *T* smaller than ours.

We now discuss the variation of different figures of merit across our devices with different *w*_S_. The data in Figure 3a and Table S1 in the Supporting Information suggest that both *V*_G,onset_^*^ and *V*_G,offset_^*^, to which we collectively refer as suppression voltages (*V*_G,supp._^*^), show a drop at highest *I*_c0_*R*_N_ ∼ 0.15 V obtained for *w*_S_ = 550 nm. Figure 3a, however, also shows that the lowest *V*_G,offset_^*^ is obtained for a device with the smallest *w*_S_ = 190 nm fabricated in our study. Therefore, we conclude that no systematic dependence of *V*_G,supp._^*^ on *I*_c0_*R*_N_ can be inferred.

A more systematic trend can be instead observed in the dependence of the power dissipated by the gate at *V*_G,offset_^*^ on *I*_c0_*R*_N_. Figure 3b in fact shows that *P*_G,offset_^*^ increases almost linearly with *I*_c0_*R*_N_, which gets in turn larger for increasing *w*_S_. We therefore conclude that a larger *P*_G,offset_^*^ is needed to trigger the GCS in wider devices. This result per se can suggest that power dissipation, which scales with *I*_leak_, can play a non-negligible role toward the GCS in our devices. We note that the data in Figure 3b–d refer to devices that have all been measured in the same cryostat. The same data of Figure 3b–d but for all devices investigated in this study (i.e., including those measured in different cryostats) are reported in Figure S5 in the Supporting Information.

Based on the significant number of devices tested, we have also explored whether there exists a minimum threshold for *P*_G,offset_^*^, above which a device always shows a GCS independently on *I*_c0_*R*_N_. To this purpose, we have calculated the ratio between *P*_G,offset_^*^ and the power dissipated by a device when this switches to the resistive state. For the latter quantity, we have considered both *P*_c0_ = *R*_N_*I*_c0_^2^ (Figure 3c) and *P*_r0_ = *R*_N_*I*_r0_^2^ (Figure 3d), where *I*_r0_ is the retrapping current measured at *V*_G_^*^ = 0. We consider both *P*_c0_ and *P*_r0_ because the *V*(*I*) characteristics of our devices are strongly hysteretic, as shown in Figure 2, and therefore a device is in a metastable state for *I*_bias_ values between *I*_c0_ and *I*_r0_. The data in Figure 2 also suggest that the difference |*I*_c0_|−|*I*_r0_|, and hence in turn *P*_c0_–*P*_r0,_ gets larger as *w*_S_ is increased.

Figure 3c–d show that, while *P*_G,offset_^*^/*P*_c0_ varies across our devices by more than 1 order of magnitude (from 6 × 10^–4^ for *w*_S_ = 550 nm to 9 × 10^–3^ for *w*_S_ = 450 nm), *P*_G,offset_^*^/*P*_r0_ shows a small deviation about an average value of ∼0.31, which seems independent of both *V*_G,offset_^*^ and *w*_S_. This finding is consistent with other reports^13^ that have shown that *P*_G,offset_^*^/*P*_r0_ is very similar across different devices, although these studies do not consider devices with large variations in *w*_S_ as we do instead here.

The data in Figure 3c also suggest that, as *I*_leak_ increases, the device switches to the normal state when *P*_G,offset_^*^/*P*_r0_ overcomes a specific threshold. Future studies can help understand whether this *P*_G,offset_^*^/*P*_r0_ threshold is set by specific physical or structural properties of the S used or, for example, by the type of substrate on which the device is made (SiO_2_/Si in all our devices), since the substrate also affects *I*_leak_ and in turn *P*_G,offset_^*^.

The small values of *P*_G,offset_^*^/*P*_c0_ measured (Figure 3c) also suggest that, unlike what one could simply conclude based on the increase of *P*_G,offset_^*^ with *I*_c0_*R*_N_ (Figure 3b), power dissipation in the form of simple Joule heating is unlikely the mechanism responsible for the GCS in this study.

### Anticorrelation between *I*_leak_ and *I*_c_

To gain further insights on how *I*_leak_ affects the GCS in our devices, we have also studied if there is any systematic correlations between *I*_leak_ and *I*_c_, meaning whether these two parameters vary independently or not.

**Figure 4:**
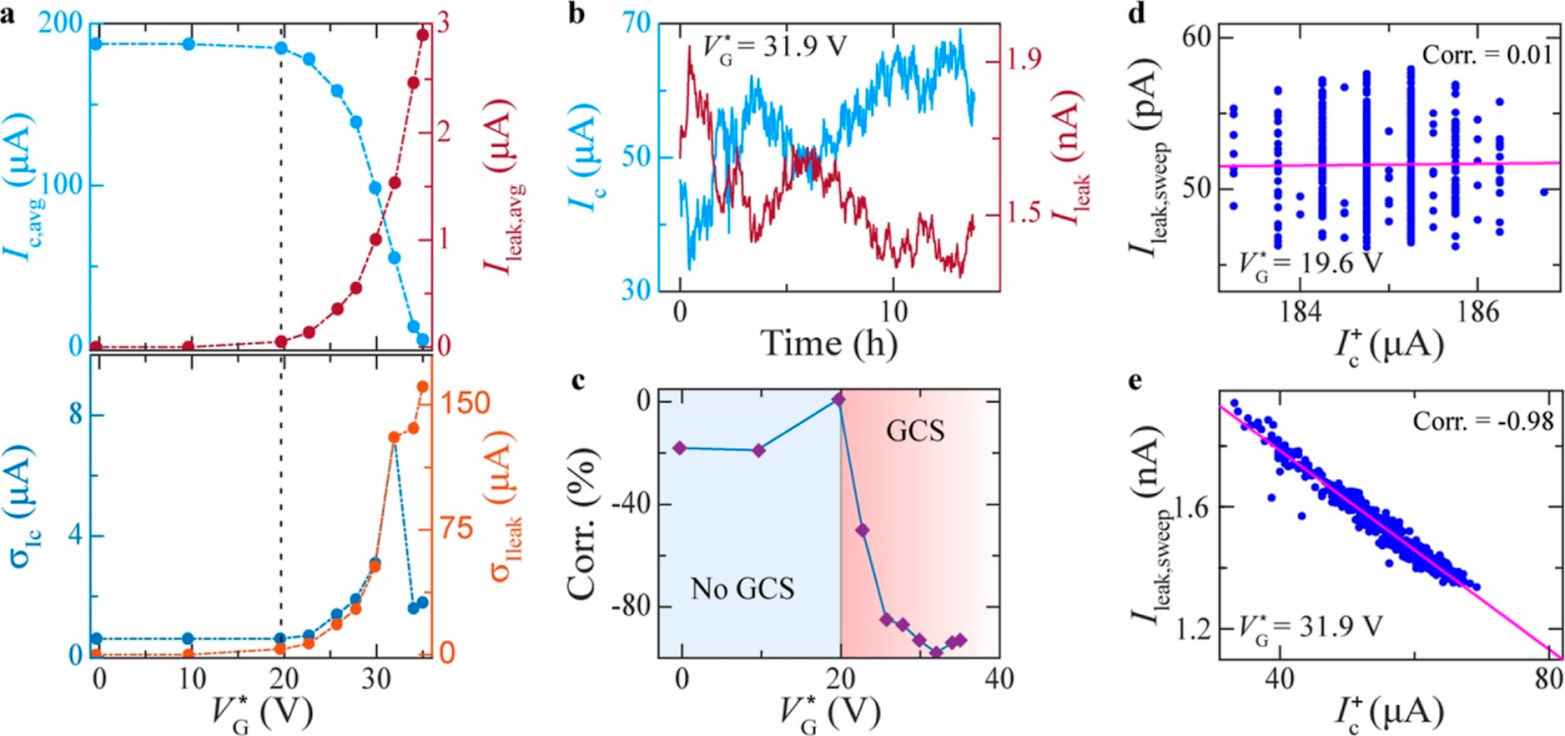
Anticorrelation between*I*_leak_ and *I*_c_. (a) Average critical current *I*_c,avg_ (top panel, left axis) and average leakage current *I*_leak,avg_ and standard deviations of the corresponding *I*_c_ distribution, σ_Ic_, (bottom panel, left axis) and *I*_leak_ distribution, σ_Ileak_, (bottom panel, right axis) as a function of the applied *V*_G_^*^. (b) Time evolution of *I*_c_ and *I*_leak_ at fixed applied *V*_G_^*^ ∼ 31.9 V. (c–e) Correlation factor *r*_*xy*_ between average *I*_leak_ per sweep, *I*_leak,sweep_, and positive *I*_c_, *I*_c_^+^, as a function of *V*_G_^*^ (c) and at specific values of *V*_G_^*^ = 19.6 V (d) and of *V*_G_^*^ ∼ 32 V (e), respectively, above and below *V*_G,onset_^*^. The magenta lines in (d,e) are the lines with equation *y* = *r*_*xy*_*x*, where *x* corresponds to *I*_c_^+^ while y to *I*_leak,sweep_. The horizontal line in (d) with *r*_*xy*_ = 0 indicates no correlation, while the diagonal line in (e) indicates almost perfect anticorrelation between the *I*_c_^+^ and *I*_leak,sweep_ sample populations. The data in this Figure are obtained for device D4 (see Table S1 in the Supporting Information).

To address this question, we have measured the average *I*_c_, *I*_c,avg_, and average *I*_leak_, *I*_leak,avg_, from a statistically significant number of measurements done at each applied *V*_G_^*^ and calculated the standard deviations of their populations which we name σ_Ic_ and σ_Ileak_, respectively. Figure 4a shows that, for *V*_G_^*^ > *V*_G,onset_^*^, *I*_leak,avg_ increases (red line), while *I*_c,avg_ decreases (blue line). On the other hand, σ_Ic_ also increases, meaning that the switching current distribution (SCD) gets wider due to GCS, as already reported,^6,17,19^ and this trend is similar for σ_Ileak_. Only for *V*_G_^*^ approaching *V*_G,offset_^*^, σ_Ic_ gets reduced^6,17,19^ (see also Figure S6 in the Supporting Information). Figure S7 in the Supporting Information shows that also the skewness of the SCD *I*_leak_ distributions vary in opposite ways for *V*_G_^*^ > *V*_G,onset_^*^.

In addition to showing opposite trends in the average amplitudes and skewness of their distributions (Figures 4a and S7 in the Supporting Information), the change in *I*_leak_ and *I*_c_ also happens concurrently and over long time scales, as shown by time-dependent evolution of their traces reported in Figure 4b at a fixed *V*_G_^*^ ∼ 31.9 V > *V*_G,onset_^*^ (and in Figure S8 in the Supporting Information for additional *V*_G_^*^ values).

The above observations therefore suggest that the amplitudes of *I*_leak_ and *I*_c_ vary simultaneously but in opposite ways, meaning that they are anticorrelated. To get a more quantitative estimate of the anticorrelation between the amplitudes of *I*_leak_ and *I*_c_, we have also determined, at a fixed *V*_G_^*^, the average *I*_leak_ measured during a single *V*(*I*) upsweep, *I*_leak,sweep_, as a function of the *I*_c_ extracted from the same *V*(*I*) curve while upsweeping the bias current (here referred to as *I*_c_^+^) and repeated this process for several *V*(*I*) sweeps. Starting from the two *I*_leak,sweep_ and *I*_c_^+^ sample populations, we determine their correlation according to the Pearson’s formula for the correlation coefficient^45^ (reported also in the Supporting Information).

The data obtained from our correlation analysis are shown for two representative values of *V*_G_^*^ in Figure 4d,e. At *V*_G_^*^ = 19.6 V, i.e. well below *V*_G,onset_^*^ ∼ 24.3 V, there is a negligible correlation ∼1% between *I*_leak,sweep_ and *I*_c_ (Figure 4d). However, when a *V*_G_^*^ ∼ 31.9 V > *V*_G,onset_^*^ is applied, the anticorrelation between *I*_leak,sweep_ and *I*_c_ reaches a value of 98% (Figure 4e). The data in Figure 4c show that this anticorrelation sets in exactly when the GCS also arises at *V*_G_^*^ > *V*_G,onset_^*^ (see also Figure S9 in the Supporting Information). The strong anticorrelation that we observe between *I*_c_ and *I*_leak,sweep_ suggests that the GCS in our Nb devices is driven by *I*_leak_.

### Effect of the Substrate and Device Training

In addition to a strong anticorrelation between *I*_leak_ and *I*_c_ (at a given applied *V*_G_^*^), our devices also show strong fluctuations in both *I*_leak_ and *I*_c_ both on a short time scale (i.e., over periods of few seconds) and on a long time scale (i.e., over several hours). We attribute the fluctuations in *I*_leak_ and *I*_c_ to electromigration and/or diffusion processes of atomic species that occur in the SiO_2_/Si substrate under an applied *V*_G_^*^. It is well-established that these processes in dielectrics like SiO_2_ can lead to the formation of metallic weak links that act as paths of low resistance for *I*_leak_ thus reducing the breakdown voltage of the dielectric.^46,47^

The same effects, however, can be achieved not only by applying a *V*_G_^*^, but also by injecting a high current through the dielectric. This process known as stress-induced leakage current (SILC) has been studied for metal-oxide-semiconductor field-effect transistors down to low temperature, where it has been reported that SILC manifests through fluctuations in *I*_leak_ occurring over periods of few seconds.^48^

In addition to SILC, current-induced stress can also lead to instabilities in *I*_leak_ associated with fluctuations of single defects under the applied *V*_G_^*^. This second effect, which can reversibly switch on/off a significant portion of the *I*_leak_ flowing through a dielectric, is also known as variable stress-induced leakage current (V-SILC).^49,50^ The main difference between SILC and V-SILC is that V-SILC can only induce an additional variation in *I*_leak_, on top of that induced by SILC.

To understand whether the fluctuations that we observe in *I*_leak_ at a fixed *V*_G_^*^ are due to SILC or V-SILC, we have performed a test, where we have probed the time evolution of *I*_leak_, *I*_leak_(*t*), at fixed *V*_G_^*^ = 27 V, while disconnecting all the instruments used for the measurement of the *V*(*I*) characteristics. This configuration allows us to suppress any possible contributions to instabilities in *I*_leak_(*t*) coming from fluctuations in *V*_G_^*^ that occur while *I*_bias_ is swept. However, since also in this measurement configuration, we still observe multiple instabilities in *I*_leak_(*t*) other than the bistability expected for a single defect, we infer that the fluctuations in *I*_leak_ originate from an ensemble of switchable defects in the SiO_2_. This is also confirmed by the fact that the power spectral density of *I*_leak_(*t*) nearly follows the trend of 1/*f*^2^-type noise (*f* being the frequency) suggesting that the *I*_leak_ values follow a Poisson distribution. This analysis reported in Figure S10 of the Supporting Information therefore suggests that *I*_leak_ most originates from V-SILC due to multilevel switching of several defects in the SiO_2_ layer (between the gate and the S constriction) under the applied *V*_G_^*^. We note that similar observations, although on different time scales, have been made for GCS devices made of semiconducting nanowires covered with Al (S) but fabricated also on SiO_2_/Si substrate like our devices.^28^ We therefore attribute the SILC and V-SILC observations to the substrate rather than to the S devices.

Having seen that the fluctuations in *I*_leak_(*t*) at fixed *V*_G_^*^ are due to V-SILC, we have also deliberately induced SILC in the SiO_2_ to study if and to which extent it affects the performance of our devices. To induce a SILC effect, on another device made on a fresh SiO_2_ substrate, we have sourced an *I*_leak_ between the gate electrode and the S constriction and monitored *V*_G_^*^, while progressively increasing *I*_leak_. Our idea here is that, as *I*_leak_ is increased, SILC should manifest and trigger a sudden change in the SiO_2_ substrate due to formation of weak links, which would in turn lead to a sudden drop of the resistance (and hence *V*_G_^*^) measured between the gate and S constriction.

**Figure 5:**
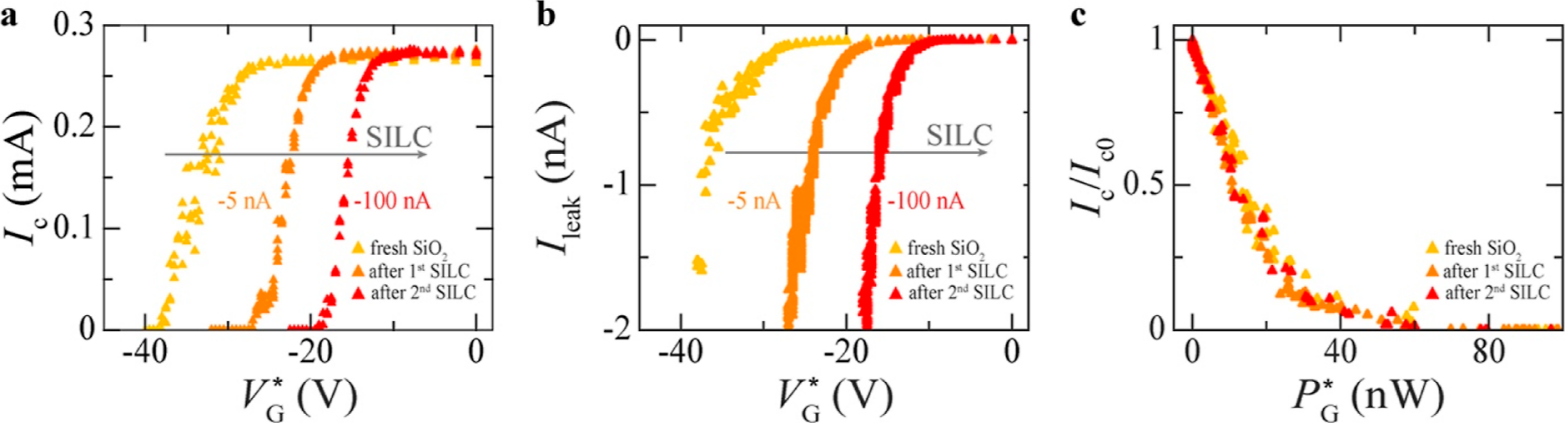
Effect of SILC on device performance. (a,b) Critical current (a) and leakage current (b) as a function of applied gate voltage, *I*_c_(*V*_G_^*^), measured after inducing subsequent SILC events through current injection between gate and S constriction. The values of the injected current triggering a SILC event are specified next to the corresponding curves. (c) Dependence of *I*_c_ (normalized to its value *I*_c0_ at *V*_G_^*^ = 0) on the power dissipated by the gate *P*_G_^*^ = *V*_G_^*^I_leak_(*V*_G_^*^) after each SILC event. The data in this figure are obtained for device D13 (see Table S1 in the Supporting Information).

Following this protocol, we have observed that a SILC-induced drop in *V*_G_^*^ indeed occurs as *I*_leak_ is increased. Once this drop is measured, we then characterize GCS in this new state of the device by measuring a full set of *I*_leak_(*V*_G_^*^) and *I*_c_(*V*_G_^*^) curves. The results of our measurements reported in Figure 5a show that the *I*_c_(*V*_G_^*^) curves measured after a SILC-related change in the SiO_2_ substrate shift toward a lower *V*_G,offset_^*^, meaning that the GCS sets in at a lower *V*_G_^*^ value.

Our results therefore suggest that SILC can be exploited in GCS devices as a resource to control their *V*_G,offset_^*^. Using this strategy, meaning by pretraining the devices through the injection of an *I*_leak_ between gate and constriction, we have achieved shifts in *V*_G,offset_^*^, Δ*V*_G,offset_^*^, up to ∼20 V (Figure 5a). This result also suggests that SiO_2_/Si is not a good substrate for the realization of GCS devices that would always work with high reliability, meaning in the same operational conditions, because SILC-related effects can occur over time and shift the working point of the GCS device. A fundamental difference between SILC and V-SILC, however, is that SILC shift the working point of the device to a new stable condition, when they occur, while V-SILC can give instabilities over short time scales, even after a new SILC-induced working point has been reached.

We note that we cannot exactly quantify how stable the new working point of a device achieved by current-induced stress training gets after subsequent cooldowns. This is because for all these devices we have intentionally increased the stress current until device failure. However, for devices that we have not intentionally stressed (apart from stress induced by the measurement current) and that we have managed to remeasure after consecutive cooldowns, we have found that the working parameters of the device (e.g., its *V*_G,offset_^*^) change within 10–20% of their original values, especially when the device is exposed to air. It is possible that this variation is due to contamination of the oxide layer in between the gate and the bridge, where defects induced by the measurement current during a certain cooldown are mostly localized and can be thus affected by contaminants once the device is exposed to air.

Although SILC results in a shift of *V*_G,offset_^*^, we find that the power suppression of *I*_c_ follows the same exact dependence on the power dissipated by the gate *P*_G_^*^ = *V*_G_^*^I_leak_(*V*_G_^*^) after each SILC event (Figure 5c). This is because, although *V*_G_^*^ decreases after a SILC event, *I*_leak_(*V*_G_^*^) increases (Figure 5b), which makes their product constant. Our observation therefore suggests that *P*_G_^*^ must always reach the same value for the GCS to occur, independently of the history of the device and previous SILC events induced therein.

## Conclusions

We have shown that three-terminal superconducting devices can exhibit GCS with very high reproducibility, which is a key result for the future development of any technological applications based on the GCS.

Starting from devices that systematically show a GCS, we also find that the effect is not limited by the width *w*_S_ of the gated superconducting constriction. Due to their large *w*_S_, our devices show high *I*_c0_*R*_N_ values (up to ∼0.25 V at 1.5 K), which suggests that values above 1 V are totally within reach at lower temperatures, especially if devices with a longer and wider constriction and/or made of a S with higher *J*_c_ or ρ_Ν_ are fabricated. The feasibility of high *I*_c0_*R*_N_ values shown by our study, together with a reduction in *V*_G,offset_, represents a significant step toward the interconnection of GCS devices and increase in their fan out.

Although our study is not aimed at revealing the mechanism underlying the GCS, our observations provide arguments against or in favor of some of the scenarios proposed to date in the literature to explain the GCS. Our findings rule out the direct field effect as the origin of the GCS in our devices because we find a strong correlation between *I*_leak_ and *I*_c_ when a GCS occurs. Similarly, the high-energy field emission scenario is not very likely to be at the origin of the supercurrent suppression in our devices because of the important role which we find that the substrate has toward the GCS properties. We can also exclude *I*_leak_-induced Joule heating, as shown by the analysis reported above.

The strong correlation observed between the suppression in *I*_c_ (i.e., the GCS) and *I*_leak_ suggests that the GCS is nevertheless *I*_leak_-induced. Therefore, among the scenarios proposed to date, we think that the scenario in better agreement with our findings is an *I*_leak_-mediated effect mediated by the substrate yet different from simple Joule heating. Whether this is a heating of the electronic system, or a perturbation of the superconducting condensate cannot be answered unambiguously on the basis of our data sets. The similarity of the critical current distribution measured by us with those reported, for example, by Basset et al.,^19^ suggests that it might be the latter effect but observed, for our devices, at a much higher level of *I*_leak_.

Although our results do not allow to draw definite conclusions on the mechanism responsible for the GCS, we have shown that, for devices made on SiO_2_, the strong influence of *I*_leak_ on the GCS can be exploited to modulate the operational *V*_G_ range of the devices. By progressively increasing *I*_leak_, the devices can be driven through several metastable transitions due to electromigration effects occurring in SiO_2_ (SILC), which reduces their *V*_G,offset_ by up to 20 V. Independently on the working point of the device and how it is shifted by previous SILC events, however, we find that the *I*_c_ suppression always occurs when the power dissipated by the gate voltage (or its relative ratio to the *P*_r0_) overcomes a specific threshold value. Although a pretraining with SILC events represents a route to modulate *V*_G,offset_ for devices made on SiO_2_, it also suggests that, for technological applications requiring devices with stable operational conditions, oxide substrates different from SiO_2_ and probably more stable against SILC effects like bare Al_2_O_3_ (sapphire)^51^ or oxide bilayer stacks (e.g., Al_2_O_3_/HfO_2_, HfO_2_/Dy_2_O_3_; refs (52 and 53)) or ternary compounds like HfAlO_*x*_ (ref (53)) should be used.

## Methods

### Sample Fabrication

Nb devices with a Ti adhesion layer have been deposited onto a 350 μm-thick (100)-oriented intrinsic Si substrate with a 300 nm-thick wet/dry/wet SiO_2_ layer on top (MicroChemicals manufacturer). Before growth of the thin films, the substrates (diced into 5 × 5 mm^2^ pieces) have been cleaned for 5 min with both acetone and isopropanole (IPA) and then blown-dry with pure N_2_.

A single 225 nm-thick layer of poly(methyl methacrylate) (PMMA) (950 PMMA A4, Kayaku) has been then spun onto then substrates and then baked on a hot plate at 180 °C for 90 s. After this step, the device geometry (Dayem bridge) has been patterned into the PMMA in a single-step electron beam lithography (EBL) process. The EBL patterning has been carried out using an acceleration voltage of 20 kV and a dose ranging between 280 and 300 μC/cm^2^. Right after exposure, the positive resist mask has been developed by dipping the samples into a methylisobutylketon (MIBK) solution (3 parts of IPA mixed with 1 part of MIBK) for 25 s.

The as-patterned samples have been then loaded into an ultrahigh vacuum (UHV) chamber with base pressure lower than 2 × 10^–8^ Torr, where the Ti adhesion layer has been sputtered by radiofrequency (RF) magnetron sputtering. For the Ti deposition, a magnetron gun power of 200 W, an Ar flow of 17 sccm and a deposition pressure of 1.5 mTorr have been used. After the Ti growth, the Nb layer has been sputtered at 300 W (17 sccm Ar flow, deposition pressure of 1.5 mTorr) using two RF magnetron guns and one DC magnetron gun simultaneously. The deposition rates both for Ti (0.055 nm/s) and Nb (0.4 nm/s) have been calibrated using atomic force microscopy.

After deposition, the devices have been immediately placed into a 50 °C hot acetone bath for lift-off for at least 3 h followed by ∼60 s of ultrasonication. Afterward the devices have been cleaned with IPA and then dried with N_2_. Before bonding, the Nb devices have been kept under N_2_ atmosphere to avoid oxidation.

### Transport Measurements

Voltage versus current, *V*(*I*), characteristics have been measured with a standard 4-point configuration using a low-noise DC current source, Keithley 6221, to inject the bias current, and a nanovoltmeter, Keithley 2182A, to measure the voltage drop across the Dayem bridge. All Dayem bridges except the ones with *w*_S_ = 190 nm have been measured in a dry inverted cryostat (Dry ICE 3K INV) with a base temperature of around 3 K. The measurement lines of this cryostat have been filtered using two-stage RC filters with a series resistance of ∼2.05 kΩ and a capacitance of 4 nF.

The devices with *w*_S_ = 190 nm have been measured in another dry cryostat (Cryogenic Ltd. manufacturer) with a base temperature of ∼1.5 K reachable with a ^4^He dip stick and of ∼300 mK reachable with a ^3^He dip stick. The lines of this cryostat have been partially filtered using a RC filter with a series resistance of 100 Ω and a capacitance of 47 nF.

The measurement temperature has been chosen to ensure a bath temperature stability of ±10 mK or better. For wires with 550 nm made with and without FIB, the bath temperature has been increased from 3.1 K (used for other devices) to 3.6–4 K (see devices D11 and D12 in Table S1 of the Supporting Information) to ensure enough cooling power and to ensure thermal stability of the measurement system, while injecting a large bias current (up to 2.6 mA).

For all devices, the leakage current has been measured using a low-noise source-measure unit, Keithley 6430, with a preamplifier connected in a two-wire configuration. For the Dry ICE 3K INV system four extra lines have been used to apply the gate voltage, which are unfiltered and shielded to measure low-leakage currents (at 70 V, these lines have more than 10 TΩ of resistance to ground). For the Cryogenic setup, the measurement lines are made of manganin twisted pairs with a double isolation of Kapton (the lines have more than 50 GΩ of resistance to ground at *V*_G_ > 50 V).

## Supporting Information Available

Table with parameters of all the devices investigated; figures with additional data on GCS devices investigated including dependence of GCS effect for FIB-cut devices, gate dependence of switching current and leakage current distributions, anticorrelation between *I*_c_ and *I*_leak_, and time evolution of V-SILC effects in SiO_2_; correlation factor (PDF)

## Notes

This work was submitted to a preprint server as Ruf, L.; Scheer, E.; Di Bernardo, A. High-performance gate-controlled superconducting switches: large output voltage and reproducibility. 2024, arXiv preprint arXiv:2404.18899. https://arxiv.org/abs/2404.18899 (accessed Jul 09, 2024).
