# Supplementary material for: High-Performance Gate-Controlled Superconducting Switches: Large Output Voltage and Reproducibility
Source: ACS Nano. 2024 Jul 26;18(31):20600–10. doi: 10.1021/acsnano.4c05910 (PMC11308776; doi:10.1021/acsnano.4c05910)
Supplement: Supplementary file 1 — nn4c05910_si_001.pdf [file nn4c05910_si_001.pdf]

Supporting Information for

High-Performance Gate-Controlled Superconducting Switches: Large Output Voltage and Reproducibility

Leon Ruf[1], Elke Scheer[1†], Angelo Di Bernardo[1§]*

1. Department of Physics, University of Konstanz, Universitätsstraße 10, 78464 Konstanz, Germany.

§ Current address: Dipartimento di Fisica "E. R. Caianiello", Università degli Studi di Salerno, via Giovanni Paolo II 132, 84084 Fisciano (SA), Italy.

[†]Email: elke.scheer@uni-konstanz.de

[*]Email: angelo.dibernardo@uni-konstanz.de

Dev id	R_N (Ω)	T_c (K)	T_m (K)	I_{c0} (μA)	I_{r0} (μA)	$V^*_{G,onset}$ (V)	$V^*_{G,offset}$ (V)	I_{leak}@ $V^*_{G,onset}$ (nA)	I_{leak}@ $V^*_{G,offset}$ (nA)	Gate sep. (nm)	Width w_S (nm)
D1	475.9	2.24	1.39	12.2	2.30	0.85	1.60	1.4	14.6	~50	190
D2	202.0	4.08	1.64	20.4	16.46	15.6	19.4	0.71	4.5	70	190
D3	203.1	3.68	3.09	15.7	8.73	24.0	32.5	0.04	n/a	< 100	~450
D4	164.1	5.08	3.10	187.0	28.00	24.3	32.5	0.17	1.6	~50	450
D5	134.6	5.14	3.14	273.0	30.07	27.5	33.6	0.21	1.3	~50	~450
D6	144.0	5.17	3.10	280.0	28.00	14.5	17.1	0.27	2.2	~50	~450
D7	142.5	5.14	3.10	256.5	30.07	26.6	34.1	0.15	1.6	~50	~450
D8	132.6	5.22	3.10	300.0	32.15	25.1	30.1	0.21	1.6	~50	~450
D9	127.9	5.34	3.16	326.2	31.5	20.6	27.1	0.14	1.5	~50	~450
D10	78.6	6.34	3.10	1245	94.00	21.2	29.7	0.22	4.0	~50	550 w/FIB cut of 90
D11	61.5	7.12	3.6	2570	136.0	5.7	9.5	0.2	25.3	~50	550
D12	61.2	7.57	4.0	2300	136.0	9.7	13.5	0.6	18	~50	550
D13	123.4	5.03	3.14	272	33.0	28.5*	37*	0.07*	0.9*	~50	550

Table S1. Parameters of the gate-controlled Nb devices investigated in this study. For each device, with identification number specified in the first column, the table reports the normal-state resistance R_N measured at 10 K, the superconducting critical temperature T_c defined as the temperature at which R_N drops by 90%, the temperature at which the device has been characterized for the GCS (T_m), the critical current (I_{c0}) and retrapping current (I_{r0}) measured at T_m without gate voltage applied, the actual gate voltage applied (i.e., corrected for the voltage drop over the wiring resistance; see main text) needed to reduce I_{c0} by 10% ($V^*_{\mathrm{G,onset}}$) and by 90% ($V^*_{\mathrm{G,offset}}$), the leakage current I_{leak} values measured at $V^*_{\mathrm{G,onset}}$ and $V^*_{\mathrm{G,onset}}$, the separation between gate and S constriction, and the width (w_S) of the S constriction. The devices reported in the first two rows have been measured in a cryostat manufactured by Cryogenic, whilst all the other devices have been measured in another cryostat manufactured by ICE Oxford (see also Methods in the main text). The symbol '*' refers to parameter values measured after I_{leak}-induced training of the device (see main text).

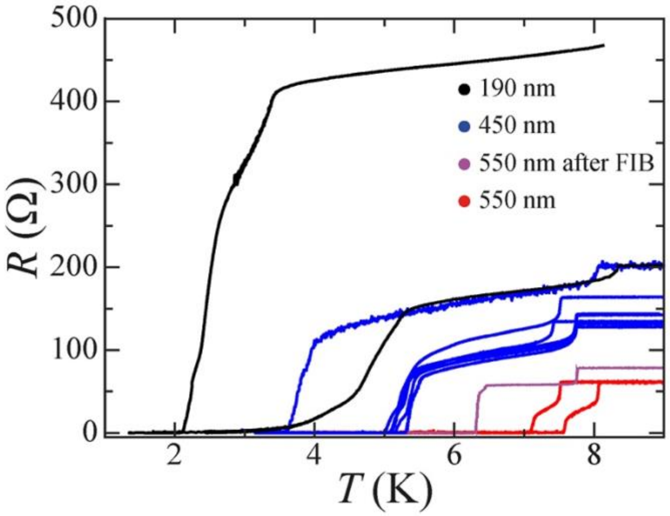

Figure S1. Low-temperature transport data of all devices. Resistance versus temperature *R*(*T*) curves measured for all Nb Dayem bridges fabricated in this study and listed in Table S1, with width w_S = 190 nm (black; 2 devices), 450 nm (blue; 7 devices); 550 nm without (red; 2 devices) and with 90-nm-wide cut made by focused ion beam on the Nb constriction opposite from the gate (purple; 1 device).

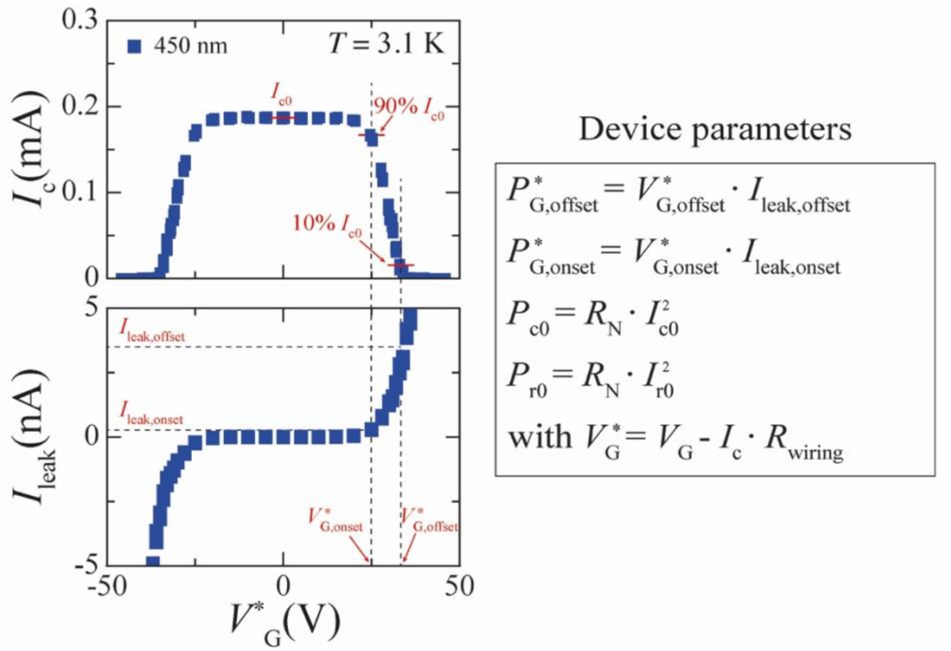

Figure S2. Definition of parameters of a GCS device. Critical current versus effective applied gate voltage, $I_c(V^*_G)$, and leakage current versus V^*_G, $I_{leak}(V^*_G)$, measured for a GCS device with width w_S = 450 nm at T = 3.1 K. From the $I_c(V^*_G)$ curve, the V^*_G values at which the I_c at V^*_G = 0 (I_{c0}) is reduced by 10% ($V^*_{G,onset}$) and by 90% ($V^*_{G,offset}$) are identified, as shown by the dashed lines in the graph. The values of I_{leak} at $V^*_{G,onset}$ ($I_{leak,onset}$) and $V^*_{G,offset}$ ($I_{leak,offset}$) are then also derived from the $I_{leak}(V^*_G)$ characteristic. The box on the right reports a list of the main device parameters that can be calculated once I_{c0}, $V^*_{G,onset}$, $V^*_{G,offset}$, $I_{leak,onset}$ and $I_{leak,offset}$ have been obtained. The meaning of these parameters and of the other variables listed is explained in the main text.

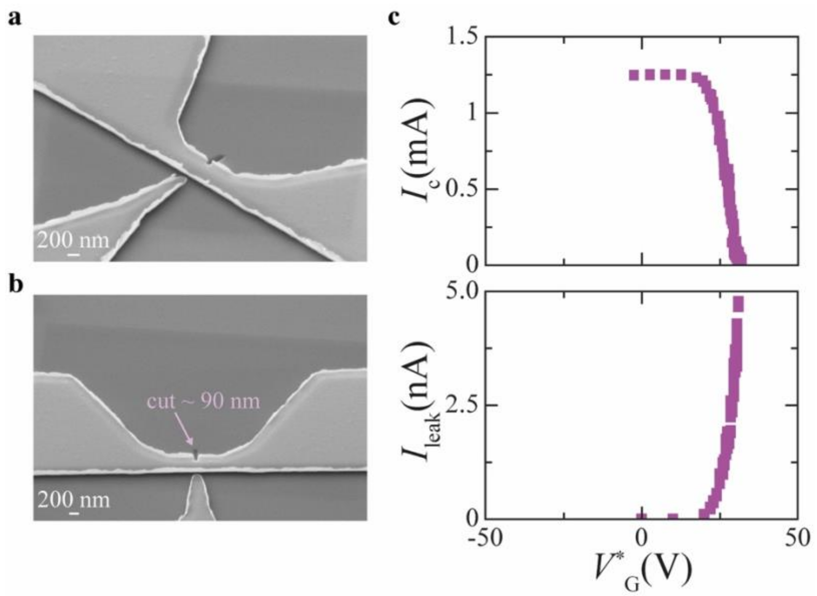

Figure S3. Characterization of a FIB-cut device. (a-b) Scanning electron micrograph images of a Nb bridge with original width w_S = 550 nm reduced by $\sim$ 90 nm using a focused ion beam with Ga^+ ions. (c) Critical current versus gate voltage $I_c(V_G^*)$ (top panel) and leakage current versus gate voltage $I_c(V_G^*)$ measured on the same device at a temperature $T \sim$ 3.1 K.

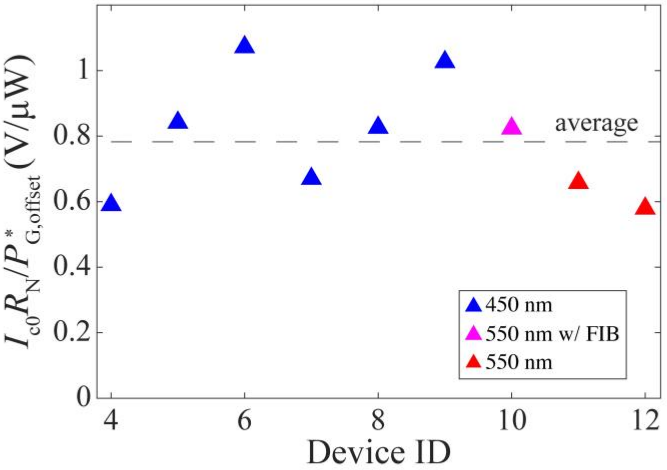

Figure S4. Variation of the characteristic voltage $I_{c0}R_N$ at $V_G^* = 0$ normalized to the power dissipated at $V^*_{G,offset}$, $P^*_{G,offset} = V^*_{G,offset} \cdot I^*_{leak,offset}$, across different devices with width w_S as specified in the figure legend. The ID for each device correspond to that reported in Table S1. All devices have been measured in the same cryostat at similar measurement temperature T_m (as specified also in Table S1).

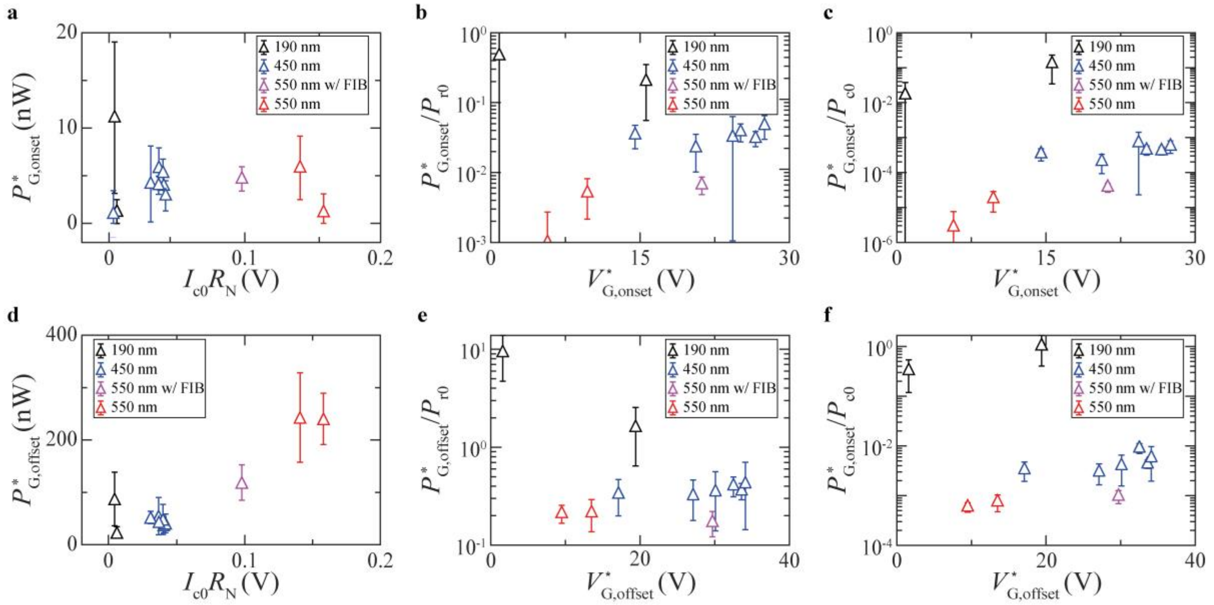

Figure S5. Performance parameters of all Nb devices studied. (a-c) Onset power dissipated by the gate $P^*_{\mathrm{G,onset}}$ as a function of the characteristic voltage $I_{c0}R_N$ (a), of the effective gate voltage $V^*_{\mathrm{G,onset}}$ for 10% I_c suppression (b), and of the effective $V^*_{\mathrm{G,offset}}$ for 90% I_c suppression (c) measured for all devices investigated with different width w_S (as specified in the legend) and at T ~ 3.1 K. (d-f) Dependence of the offset power dissipated by the gate $P^*_{\mathrm{G,offset}}$ on the same parameters $I_{c0}R_N$ (d), $V^*_{\mathrm{G,onset}}$ (e), $V^*_{\mathrm{G,onset}}$ (f) for the same devices reported in (a-d) (see legends for w_S values) and measured at the same T ~ 3.1 K.

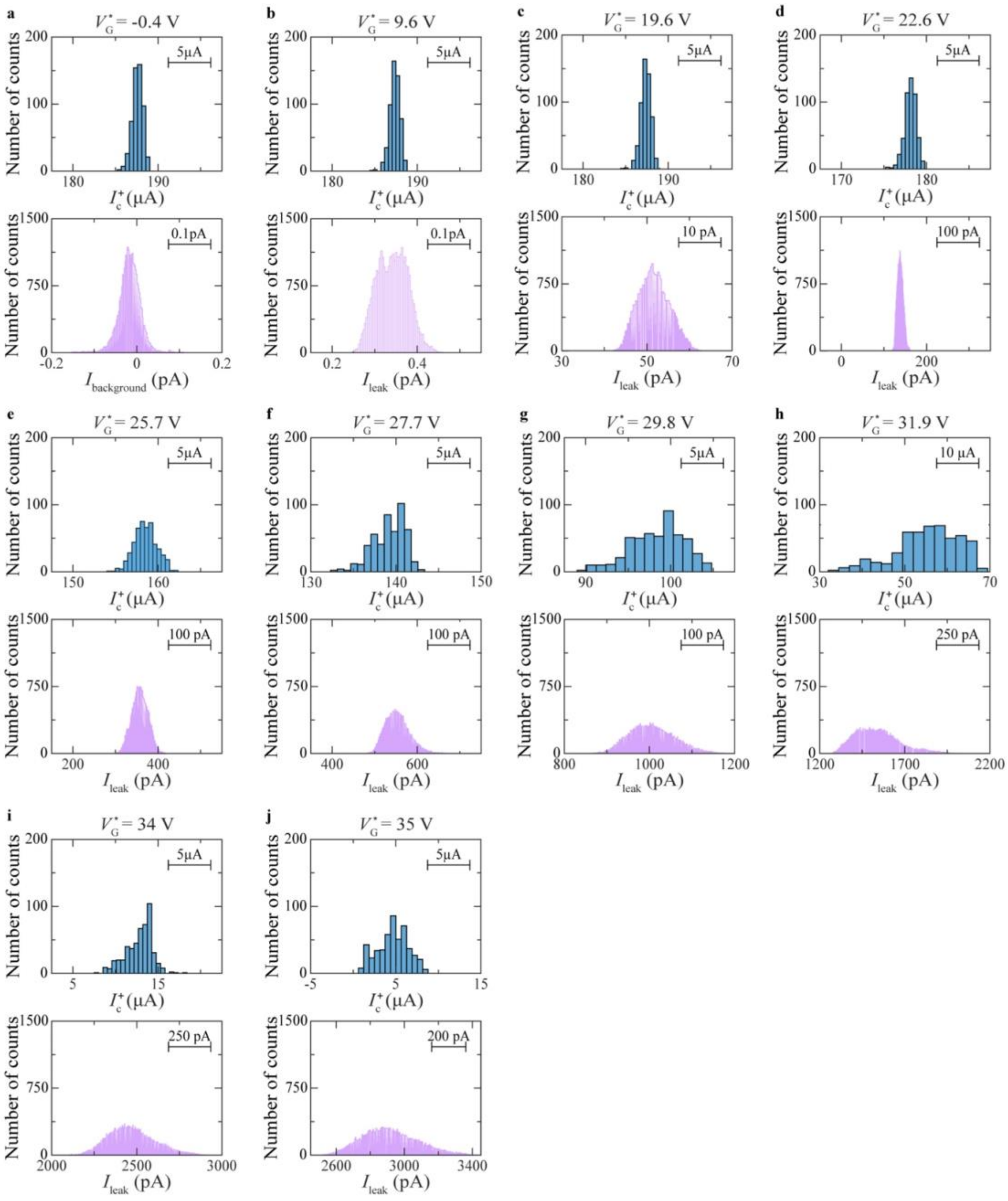

Figure S6. Gate dependence of switching current and leakage current distributions. (a-j) Distributions of the positive critical current I_c^+ (top part of the panel) and of the leakage current I_{leak} (bottom part of the panel) measured for the same device shown in Figs. S8 and S9 at different gate voltage V_G^* (V_G^*values reported on top of each panel) and temperature $T \sim 3.1$ K. I_c^+ has been measured whilst upsweeping the bias current from 0 to positive values.

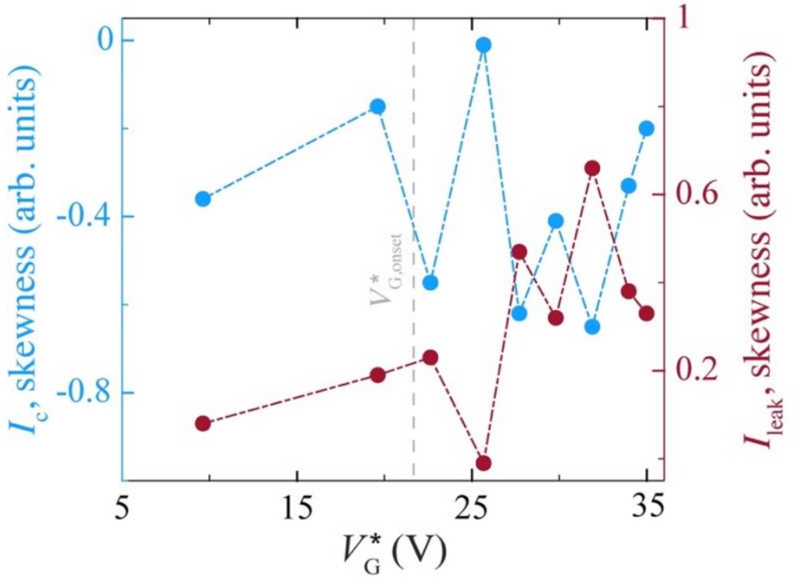

Figure S7. Anticorrelation between leakage and critical current distributions. Dependence of the skewness of the distributions of the critical current I_c (left axis; blue curve) and of the skewness of the leakage current I_{leak} (right axis; red curve) measured for a gated Nb device as a function of the effective gate voltage V_G^* applied. The two curves show a clear anticorrelation concomitant with the occurrence of the GCS for $V_G^* > V_{G,onset}^*$ (dashed gray line).

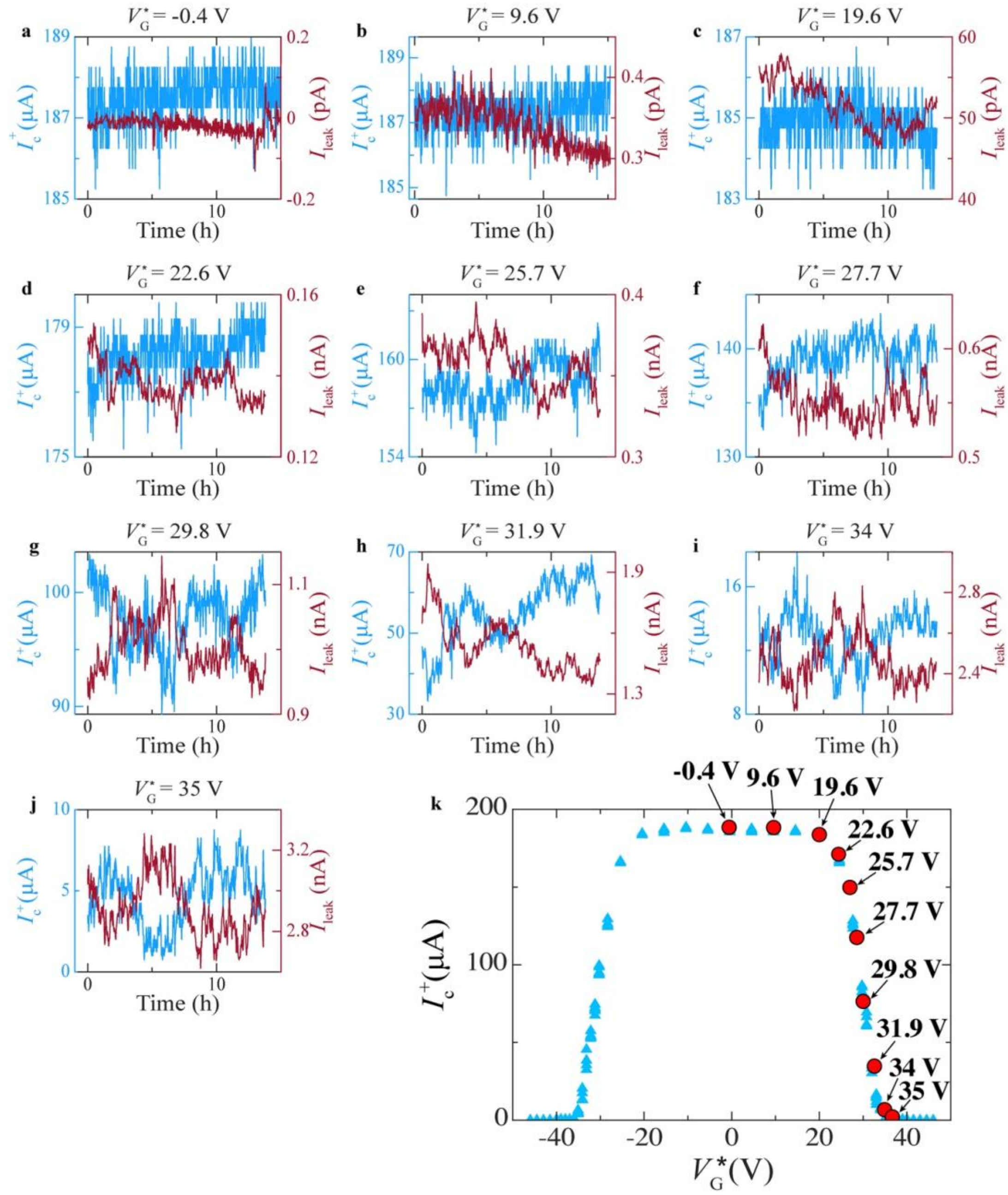

Figure S8. Time evolution of I_c^+ and I_{leak}. (a-j) Positive critical current I_c^+ and leakage current I_{leak} measured as a function of time at fixed applied gate voltage V_G^* (V_G^* values indicated on top of each panel) and temperature $T \sim 3.1$ K. At a given time t, I_c^+ is obtained from the voltage versus current $V(I)$ characteristics measured by upsweeping the bias current I from 0 to positive values. (k) $I_c^+(V_G^*)$ curve showing the observation of a GCS and reporting the V_G^* values (marked with red circles) at which the measurement data in panels from (a) to (j) have been obtained in the same device.

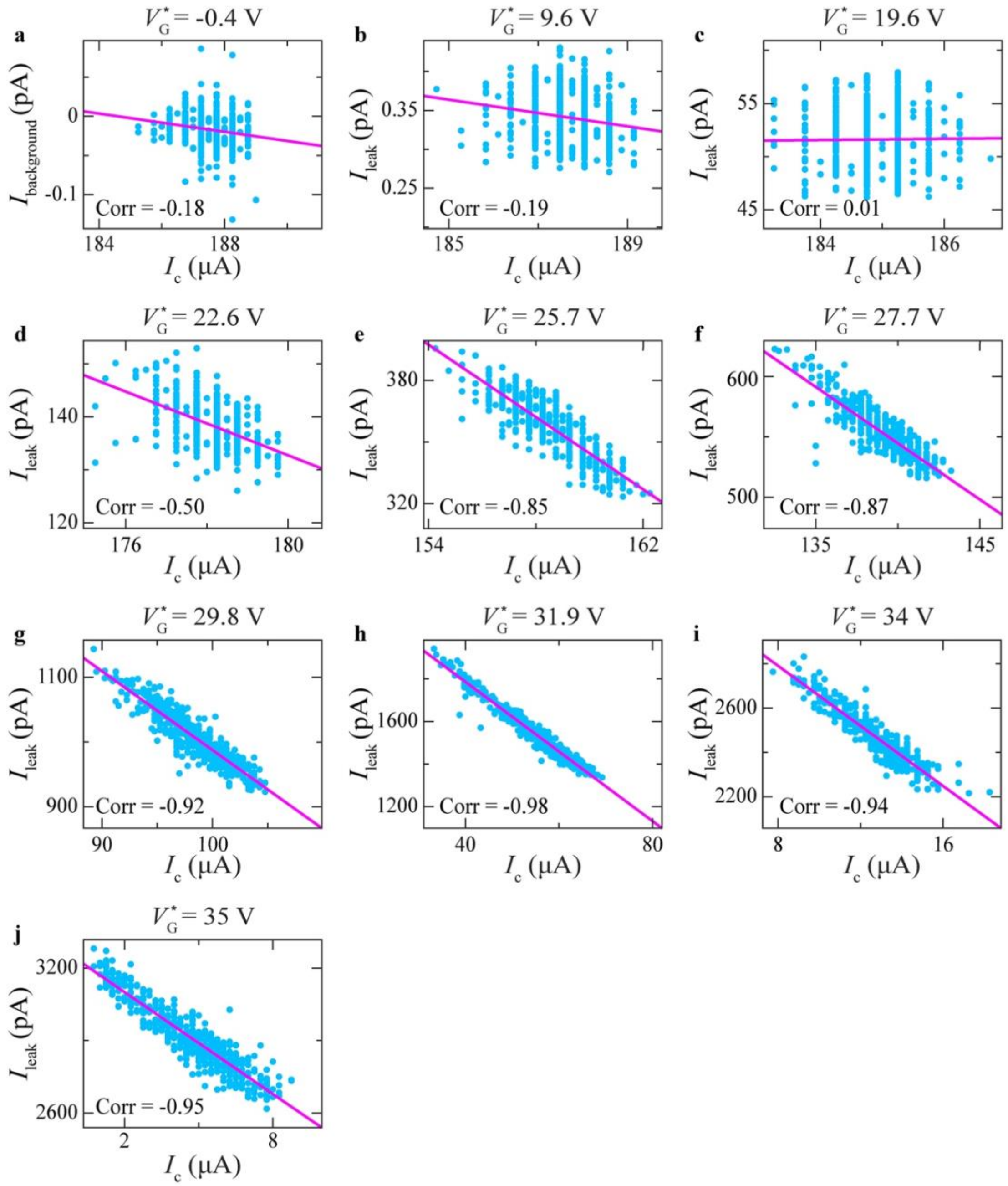

Figure S9. Anticorrelation between I_c and $I_{leak,sweep}$. (a-j) Average leakage current per sweep $I_{leak,sweep}$ as a function of the positive critical current I_c^+ measured for the same device shown in Figs. S6 and S8 at different gate voltage V_G^* (V_G^*values reported on top of each panel) and temperature $T \sim 3.1$ K. For each panel, the applied V_G^* is the same as that of the panel labelled with the same letter in Figs. S6 and S8, and the correlation factor obtained is reported in the bottom-left corner. For panel (a), the noise on I_{leak} due to the instrumental setup at $V_G^* = 0$ is reported.

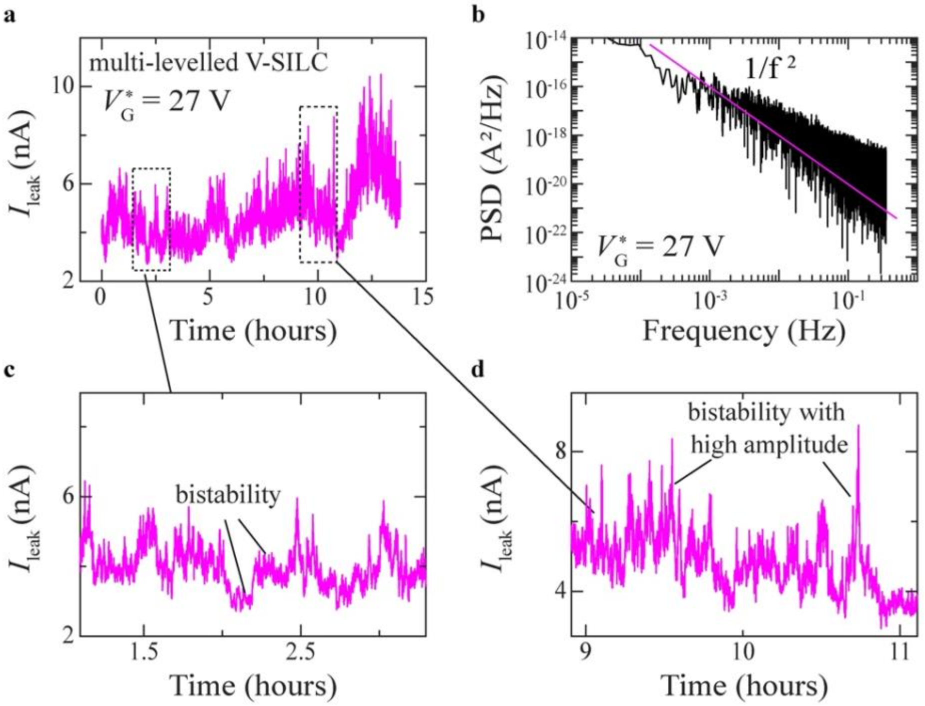

Figure S10. Instabilities in leakage current and relation to SiO_2/Si substrate. (a) Leakage current measured over time I_{leak} for a Nb device under an applied gate voltage V_G^* = 27 V above the onset voltage of the GCS for the device. (b) Power spectral density (PDS) corresponding to the signal in (a) showing trend close to $1/f^2$-type noise. (c-d) I_{leak} signal in (a) over selected time periods (indicated by dashed boxes) showing multiple instabilities consistent observed for multi-levelled variable stress-induced leakage current (V-SILC) in SiO_2, in addition to bistabilities with small (c) or large (d) amplitudes.

Supplementary Text. The correlation factor reported in Figs. 4d,e and in the Supplementary Fig. S9 represents the Pearson correlation coefficient r_{xy} originally defined in ref.[45] of the main manuscript and given by the formula:

$$r_{xy} = \frac{\sum_{i=1}^{n}(x_i - \bar{x})(y_i - \bar{y})}{\sqrt{\sum_{i=1}^{n}(x_i - \bar{x})^2}\sqrt{\sum_{i=1}^{n}(y_i - \bar{y})^2}},$$

where *n* is the sample size, x_i and y_i represent the individual sample values, and $\bar{x}$ and $\bar{y}$ are the averages of the x_i and y_i values. For the data reported in the above-listed Figures, the x_i and y_i values correspond to the measured I_c^+ and $I_{\text{leak,sweep}}$ values, respectively. The value of r_{xy} can vary between -1 (for perfect anticorrelation) to +1 (for perfect correlation). When $r_{xy} = 0$, this means that there exists no correlation between the two sample populations.
